# Genome-wide detection of m6A-associated SNPs in atrial fibrillation pathogenesis

**DOI:** 10.3389/fcvm.2023.1152851

**Published:** 2023-05-26

**Authors:** Yan Huang, Yuqian Tan, Yuan Yao, Linglong Gu, Liusong Huang, Tao Song

**Affiliations:** ^1^ Department of Cardiology, Renmin Hospital of Wuhan University, Wuhan, China; ^2^ Cardiovascular Research Institute of Wuhan University, Wuhan, China; ^3^ Hubei Key Laboratory of Cardiology, Wuhan, China; ^4^ Department of Health Toxicology, Key Laboratory for Environment and Health, School of Public Health, Tongji Medical College, Huazhong University of Science and Technology, Wuhan, China; ^5^ College of Software Engineering, Maanshan Teacher's College, Maanshan, China

**Keywords:** N6-methyladenosine methylation, atrial fibrillation, single nucleotide polymorphism, genome-wide association study (GWAS), m6A modification

## Abstract

**Objective:**

N6-Methyladenosine (m6A) modification is of great importance in both the pathological conditions and physiological process. The m6A single nucleotide polymorphisms (SNPs) are associated with cardiovascular diseases including coronary artery disease, heart failure. However, it is unclear whether m6A-SNPs are involved in atrial fibrillation (AF). Here, we aimed to explore the relationship between m6A-SNPs and AF.

**Method:**

The relationship between m6A-SNPs and AF was evaluated by analyzing the AF genome-wide association study (GWAS) and m6A-SNPs annotated by the m6AVar database. Further, eQTL and gene differential expression analysis were performed to confirm the association between these identified m6A-SNPs and their target genes in the development of AF. Moreover, we did the GO enrichment analysis to figure out the potential functions of these m6A-SNPs affected genes.

**Result:**

Totally, 105 m6A-SNPs were identified to be significantly associated with AF (FDR < 0.05), among which 7 showed significant eQTL signals on local genes in the atrial appendage. By using four public AF gene expression datasets, we identified genes *SYNE2*, *USP36*, and *THAP9* containing SNPs rs35648226, rs900349, and rs1047564 were differentially expressed in AF population. Further, SNPs rs35648226 and rs1047564 are potentially associated with AF by affecting m6A modification and both of them might have an interaction with RNA-binding protein, PABPC1.

**Conclusion:**

In summary, we identified m6A-SNPs associated with AF. Our study provided new insights into AF development as well as AF therapeutic target.

## Introduction

Atrial fibrillation (AF) is the most common arrhythmia, which can lead to severe stroke and heart failure and carries high risk of morbidity and mortality. AF affects millions of people worldwide with a prevalence of 2%–4% in the general population (1). The risk of developing AF is intimately associated with factors as diabetes, hypertension, heart failure, and smoking as well as alcohol et al. (2). Despite of over 100 years of research, the fundamental mechanisms of AF is not totally well known. AF results from interactions between triggers which are responsible for its initiation, and the substrate that is responsible for its perpetuation. Haïssaguerre et al. (3) first demonstrated in human patients that the atrial sleeves in the pulmonary veins harbor the vast majority of the ectopic electric triggers that initiate AF. His results have been confirmed by the following researchers (4). Since then, electrical isolation of the pulmonary veins became universal therapeutic means to prevent AF. Mechanisms underlying the perpetuation of AF are still debated. Multiple wavelets and localized (focal or reentrant) sources are largely accepted to drive AF. Besides, electrical remodeling, structural remodeling, genetic predisposition, and neuro-humoral contributors make the interactions between initiation and perpetuation more complex (5). AF appears to be highly heritable. Nearly one fifth of AF is familial, which suggests a genetic predisposition. To date, GWAS studies have identified a dozen of genetic loci associated with AF. Most of the gene mutation sites related to AF are from ion channel gene mutations, such as *SCN5A, KCNQ1, ABCC9,* etc., but non-ion channel gene mutations have also been found in patients with AF, including *NPPA, TBX5, MYL4* genes (6). GWAS has also shown single-nucleotide polymorphism (SNP) is strongly associated with susceptibility to AF (7, 8).

N6-methyladenosine (m6A) modification, methylation of the sixth nitrogen atom on the RNA molecule adenosine, is the most abundant internal modification of messenger RNA (mRNA) in eukaryotic cells, which was also detected in transfer RNA (tRNA), ribosome RNA (rRNA) as well as non-coding RNA (ncRNA). m6A modification mainly occurs in the 3′-untranslated regions and nearby the stop codons of mRNAs (9). The process of m6A methylation is dynamic and reversible and is regulated by m6A methylation regulators including “writers”, “erasers” and “readers”. m6A writers are composed of RNA methyltransferase complex, such as methyltransferase like 3 (METTL3), methyltransferase like 14 (METTL14) as well as Wilm's tumor 1-associated protein (WTAP) (10). m6A erasers are demethylases including Fat mass and obesity-associated protein (FTO) and α-ketoglutarate-dependent dioxygenase alk B homolog 5 (ALKBH5) (11, 12). m6A readers are RNA-binding proteins (RBPs) that regulate the mRNA processing and metabolism containing the YT521-B homology (YTH) domain family (YTHDF1, YTHDF2, YTHDF3, YTHDC1, YTHDC2), ELAV-like protein 1 (ELAVL1), insulin-like growth factors (IGF2BP1, IGF2BP2 and IGF2BP3), et al. (13). m6A mainly occurs within a highly-conserved consensus motif RRACH, in which R represents A or G, and H represents A, C or U (14). m6A methylation regulates the splicing, transport, storage, decay and translation of mRNAs, and plays crucial roles in various cellular pathways and processes such as cell differentiation, development and metabolism (14).

Evidences have shown m6A modification affects gene expression and plays an essential role in heart development and pathophysiological process of cardiovascular diseases including myocardial infraction (15), heart failure, myocardial hypertrophy (16), and heart regeneration (17). Wang et al. reported WTAP promoted myocardial ischemia-reperfusion injury through promoting endoplasmic reticulum stress and cell apoptosis by regulating m6A modification of the activating transcription factor 4 (ATF4) mRNA (15). Berulava et al. found m6A level is altered in heart hypertrophy and heart failure and m6A RNA methylation changes lead to changes in protein abundance, unconnected to mRNA levels, which contribute to heart failure progression (18). In another study, the FTO protein expression is reduced in failing heart, which increases m6A in RNA and decreases cardiomyocyte contractile function (19). Moreover, recent studies have confirmed that single-nucleotide polymorphisms (SNPs) can affect the m6A modification by changing the RNA sequence of the target site and key flanking nucleotides and these SNPs have been suggested to be associated with various diseases including coronary artery disease, ischemic stroke and cancer (20–22). However, it is unclear whether there is relationship between m6A-SNP and arrhythmia. Here, we investigated the potential roles of m6A-SNP and AF by using the public GWAS and m6Avar database, which may provide novel insights to AF development.

## Materials and methods

### AF associated m6A-SNPs selection

**Figure 1:**
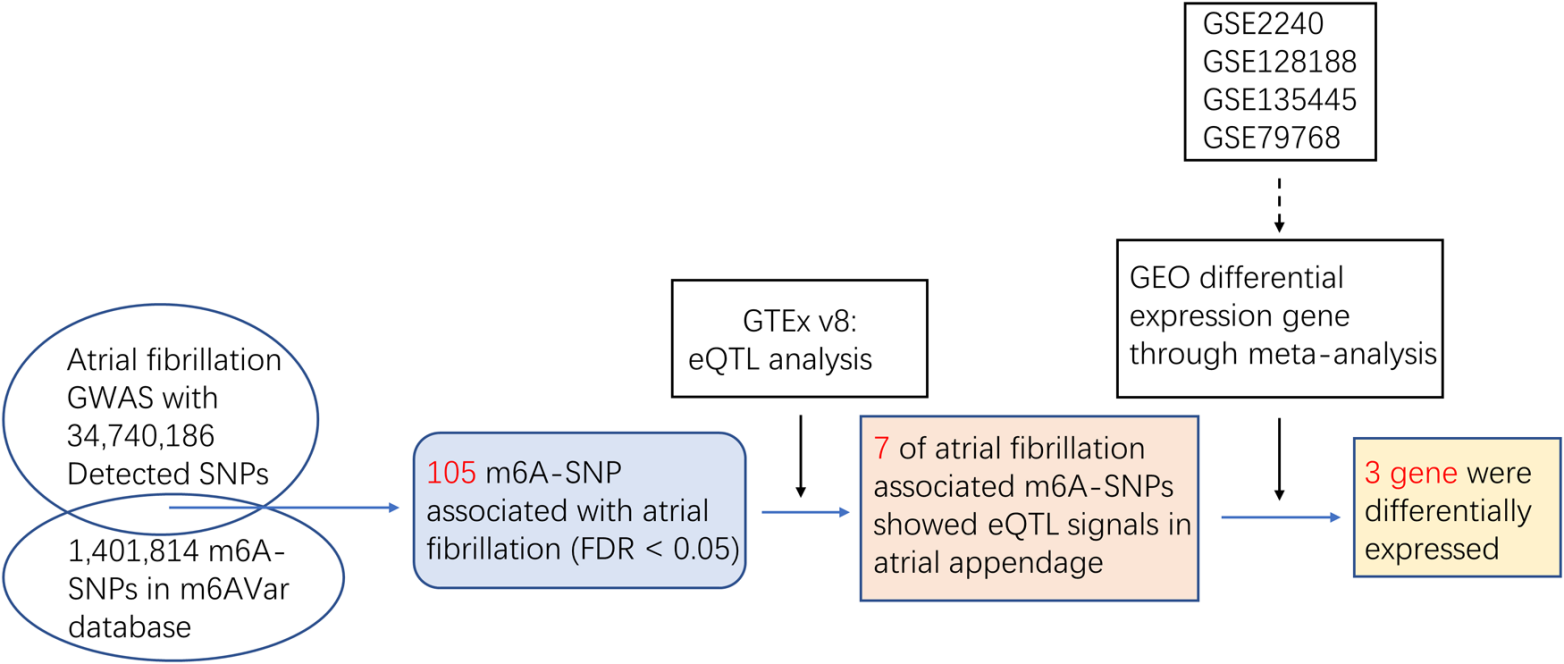
Flow chart of the study design and the main results.

We first explored the effects of m6A-SNPs on AF using the published GWAS data (nielsen-thorolfsdottir-willer-NG2018-AFib-gwas-summary-statistics.tbl), which included 60,620 AF cases and 970,216 controls with 34,740,186 SNPs in European-American population. To identify the m6A-SNPs in these 34,740,186 SNPs, we obtained the m6A-SNPs from the m6AVar database (http://rmvar.renlab.org/download.html). This database contains three confidence levels: 217,241m6A-SNPs are in high confidence level, 506,235 m6A-SNPs are in medium confidence level, 678,338 m6A-SNPs are in low confidence level. We therefore tested the association between these m6A-SNPs and the risk of AF (Figure 1).

### Expression quantitative trait loci (eQTL) analysis of m6A-SNPs associated with AF risk

We performed the eQTL analysis to explore the possible functions for the AF associated m6A-SNPs in atrial appendage from European-American subjects by using the GTEx V8 database (https://gtexportal.org/home/), through downloaded significant variant gene pairs in GTEx, which contained 1,475,414 significant variant gene pairs. The nominal *p*-value threshold is for calling a variant gene pair signature for the gene. HaploReg browser (https://www.encodeproject.org/software/haploreg/), which aggregated eQTL data from 13 studies carried out in different human cells and tissues is used to validate whether the eQTL signal of the identified m6A-SNP is displayed. In addition, through HaploReg, potential effects on local gene expression and function annotation of the identified m6A-SNPs were analyzed to determine their possible roles in transcriptional regulation. This eQTL analysis was to find m6A-SNPs showed eQTL signals and get potential functional evidence of affecting AF for these SNPs.

### Gene ontology (GO) enrichment analysis and quality control and association analysis

GO database is a powerful system for gene annotation. Through GO analysis of identified AF-related genes, with which the m6A-SNPs showed eQTL signals, we can classify these genes according to different functional annotation and figure out which genes associated with AF are related to the functional changes. Gene list enrichments are identified in the following ontology categories: DisGeNET. The Metascape tools (https://metascape.org/gp/index.html#/main/step1) were used to do GO enrichment analysis and Quality Control and Association Analysis.

### Differential expression analysis

We further explored whether the expression of the AF-related m6A-SNPs regulated genes were related to AF. We therefore detected if the genes in which AF-associated m6A-SNPs located were expressed differently between AF population and healthy controls. GSE128188, GSE2240, GSE135445 and GSE79768 datasets were used for analyzing the different expressions between healthy controls and AF population. We used the online website network analyst (https://www.networkanalyst.ca/) to meta-analyze the differential genes of those datasets. The GEO dataset (http://www.ncbi.nlm.nih.gov/geo) collects microarray and sequencing information of gene expressions for various diseases. The DEmRNAs with combined *p *< 0.05 were considered to be significant in differential expression analysis.

### The m6A modifications prediction

To examine whether these AF-related m6A-SNPs play a role in m6A modification and predict its changes, a computational predictor of m6A modification site named sequence-based RNA adenosine methylation site predictor (SRAMP), was used to perform the analysis (23). After the reference sequence and changed sequence were input into SRAMP, the m6A modification sites near the m6A-SNP will be detected and the confidence of m6A modifications was displayed as well. Meanwhile, the potential functions of selected m6A-SNPs were analyzed by querying UCSC database.

## Results

### Identification of m6A-SNPs associated with AF risk

**Figure 2:**
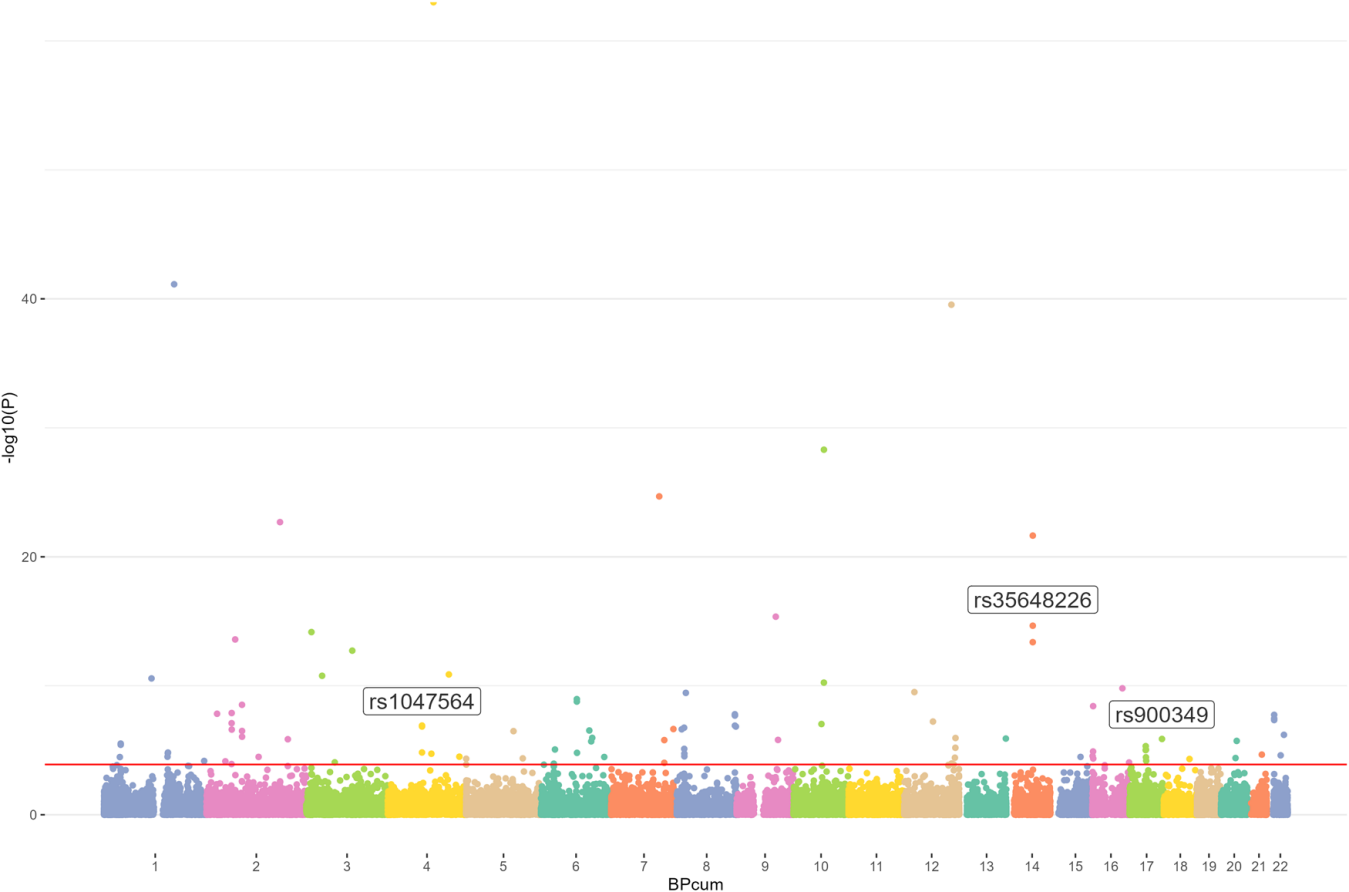
Genome-wide results for the relationship between m6A-SNPs and AF. Manhattan plot showed105 m6A-SNPs were associated with AF (FDR < 0.05). SNPs that may affect m6A modification of AF were annotated.

We first used the AF GWAS and the m6Avar databases to find out the intersection and selected out these m6A-SNPs associated with AF. In this study, we analyzed 34,740,186 SNPs from the GWAS for atrial appendage and 1,401,814 m6A-SNPs from the m6Avar database, identifying a total of 42,711 m6A-SNPs and a Manhattan plot containing these m6A-SNPs was generated. Furthermore, we identified 105 m6A-SNPs that were significantly associated with AF risk with False Discovery Rate (FDR) was less than 0.05 (Figures 1, 2).

### The eQTL analysis identified target genes of significant m6A-SNPs

We then tried to figure out the potential roles underlying the 105 identified m6A-SNPs related to AF. Since m6A-SNPs may have effect on RNA modification as well as the risk of AF, they may act through changing the expression of local genes. We further investigated whether these AF-associated m6A-SNPs were related to the expression levels of local genes by using eQTL analysis. As a result, 7 of the 105 identified AF associated m6A-SNPs showed significant eQTL signals in atrial appendage using GTEx database with threshold: nominal *p*-values < the nominal *p*-value threshold (Table 1). Besides, all the 7 SNPs were loss of function variants.

**Table 1:** Seven m6A-SNPs of seven genes associated with atrial fibrillation shows eQTL signals.

SNP ID	Chr	Position	M6A_ID	Gene	Gene_region	Confidence level	Modification function	A1	A2	Effect A2	FDR
rs35648226	14	64,21,9407	RMVar_ID_980070	SYNE2	CDS	Prediction:(Low)	Functional Loss	T	G	0.0595	9.47E-12
rs2815301	16	19,54,717	RMVar_ID_1018847	RPL3l	intron	Prediction:(Low)	Functional Loss	T	C	0.0504	6.26E-06
rs1007654	17	39,95,5101	RMVar_ID_727147	GSDMA	intron	m6A-Label-seq:(High)	Functional Loss	A	G	0.0283	1.65E-02
rs900349	17	78,83,6051	RMVar_ID_1081501	USP36	intron	Prediction:(Low)	Functional Loss	T	C	−0.0323	9.49E-04
rs28369993	2	12,66,84151	RMVar_ID_345342	GYPC	intron	MeRIP-seq:(Medium)	Functional Loss	A	G	0.1231	1.60E-02
rs1047564	4	82,91,9753	RMVar_ID_1330493	THAP9	3′UTR	Prediction:(Low)	Functional Loss	A	G	-0.0354	1.32E-04
rs1226590	9	10,05,78087	RMVar_ID_1468132	CAVIN4	5′UTR	Prediction:(Low)	Functional Loss	A	G	−0.0325	1.09E-03

### GO enrichment analysis demonstrated potential functions

**Figure 3:**
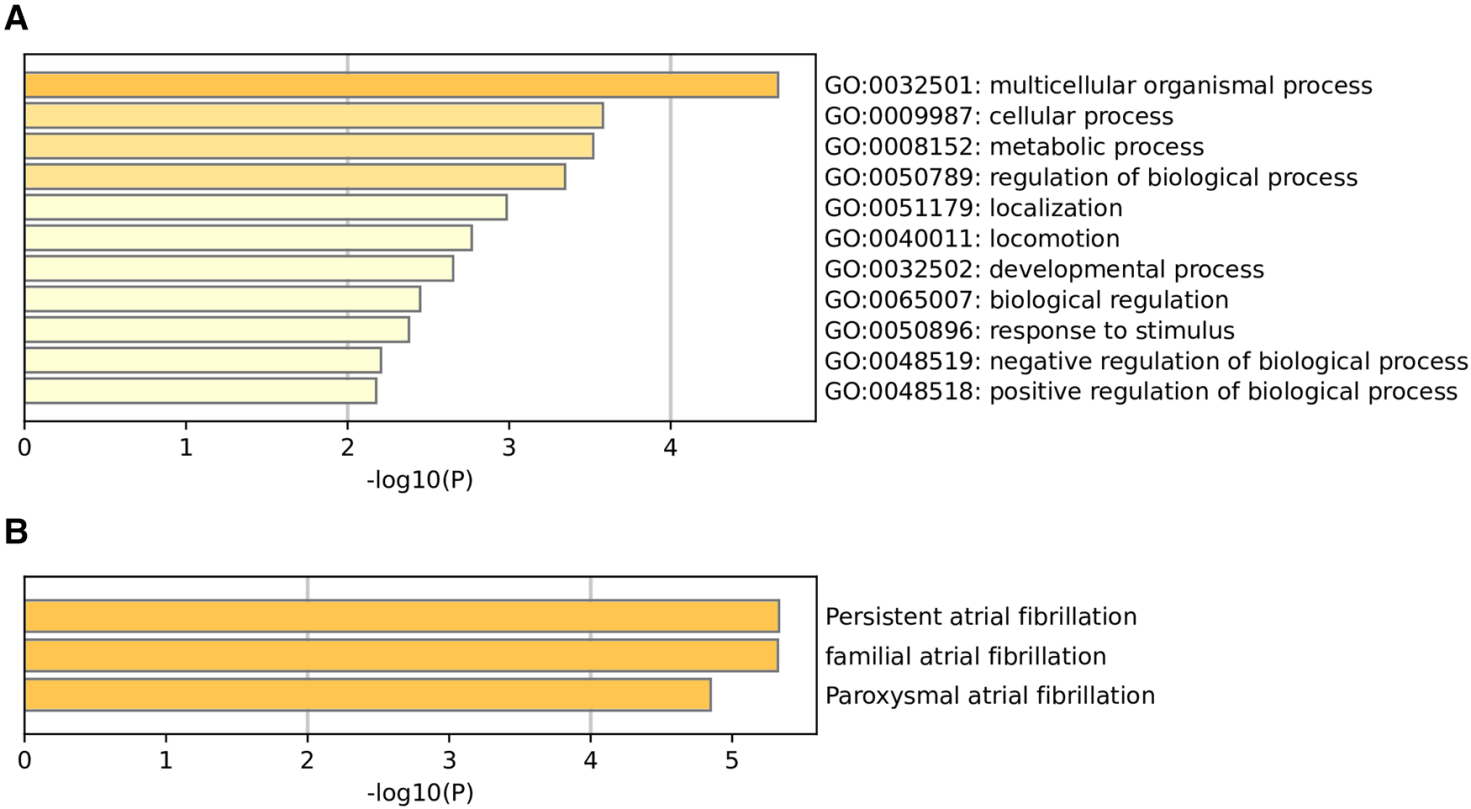
Go enrichment analysis (**A**) and quality control and association analysis (**B**) was used to elucidate the potential functions of genes corresponding to identified m6A-SNPs.

To functionally annotate the identified SNPs, we carried out GO enrichment analysis. The GO enrichment analysis suggested that the biological functions of these genes of 105 m6A-SNPs include multicellular organismal process (GO:0032501), cellular process (GO:0009987), metabolic process (GO:0008152), regulation of biological process (GO:0050789), localization (GO:0051179), locomotion (GO:0040011), developmental process (GO:0032502), biological regulation (GO:0065007), response to stimulus (GO:0050896), negative regulation of biological process (GO:0048519), positive regulation of biological process (GO:0048518) (Figure 3A). As for quality control and association analysis of 7 genes regulated by 7 m6A-SNPs with eQTL signals, the top few enriched clusters (persistent atrial fibrillation:C2585653, familial atrial fibrillation:C3468561, paroxysmal atrial fibrillation:C0235480) are shown in the Figure 3B.

### The genes expression analysis of the target genes of identified m6A-SNPs

**Figure 4:**
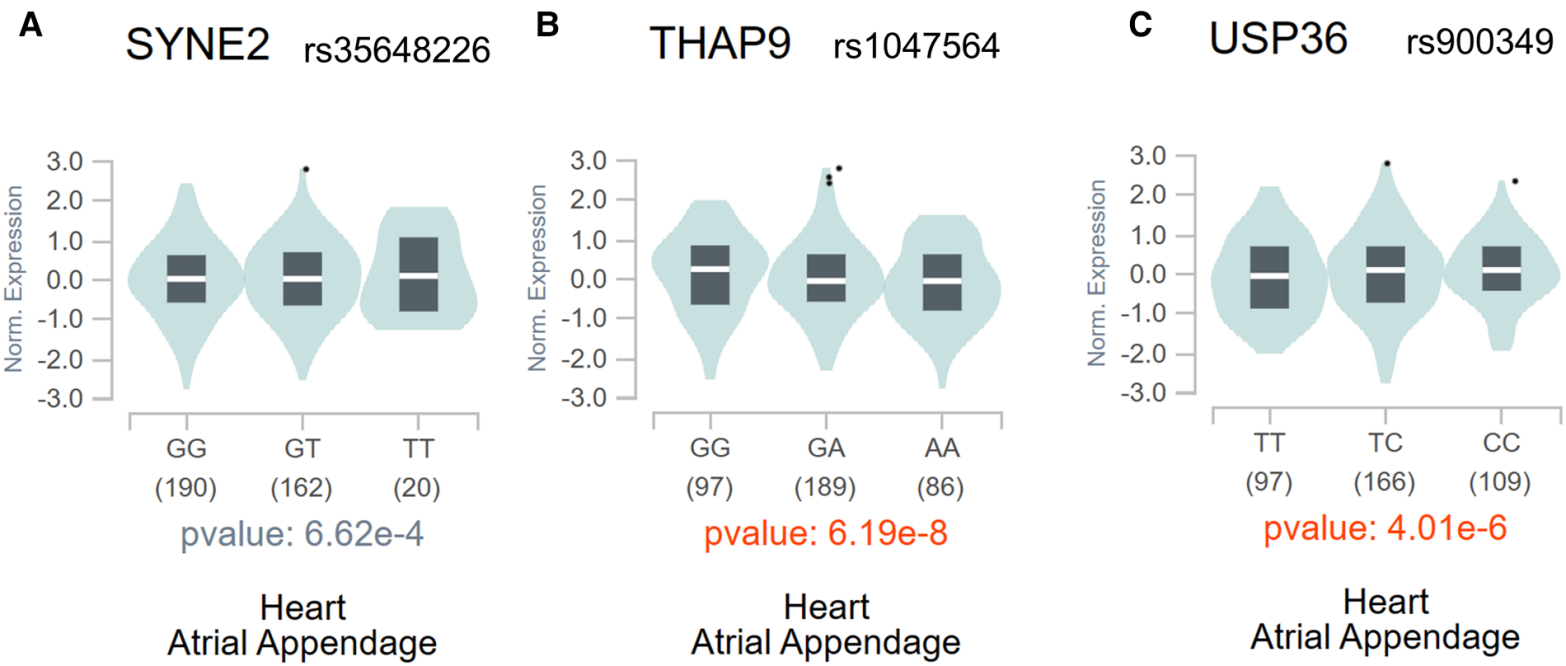
The violin plots of the eQTLs of *SYNE2* (**A**), *THAP9* (**B**), and *USP36* (**C**) in the atrial appendage in the whole population through the GTEx V8 eQTL calculator.

The above analysis found a total of 7 m6A-SNPs showing eQTL signals. In order to analyze the mRNA expression levels of the local genes containing these SNPs, we further compare the mRNA expression levels between AF cases and healthy ones in four RNA expression datasets (GSE128188, GSE2240, GSE135445 and GSE79768), consisting 40 patients with atrial fibrillation, 48 controls and 8,636 genes. The meta-analysis results showed all the 3 genes (*SYNE2*, *USP36* and *THAP9*) corresponding to 3 m6A-SNPs (rs35648226, rs900349 as well as rs1047564) were differentially expressed (combined *p *< 0.05) (Table 2). This result suggested these m6A-SNPs might also have influence on AF risk by affecting the expression of local genes. The regulatory functions of the 3 m6A-SNPs, shown in Table 2, was performed to determine their possible roles in transcriptional regulation by HaploReg. SNPs with nominal *p*-values < the nominal *p*-value threshold were selected in European-American subjects and the GTEx V8 eQTL Calculator was used to further prioritize the eQTLs of *SYNE2*, *USP36* and *THAP9* in the atrial appendage (Figure 4).

**Table 2:** 3 genes of m6A-SNPs were differentially expressed in European-American subjects and their annotations by HaploReg browser.

SNP ID	CHR: POS: 38	DNAse	Motifs	Selected eQTL hits	Pval_nominal (GTEx V8)	Pval_nominal_ threshold	Gene	Combined p-value
rs35648226	14:64219407	HRT	12 altered motifs	14 hits	3.18E-06	5.71E-05	SYNE2	1.38E-03
rs900349	17:78836051	BLD, BLD, SKIN	HNF1, Pax-4, SP2	24 hits	1.83E-05	3.77E-05	USP36	4.74E-03
rs1047564	4:82919753	GI, GI	CTCF, DEC, ZEB1	10 hits	9.44E-08	9.74E-05	THAP9	1.03E-03

### *SYNE2* (rs35648226) and *THAP9* (rs1047564) may affect m6A modification in AF

**Figure 5:**
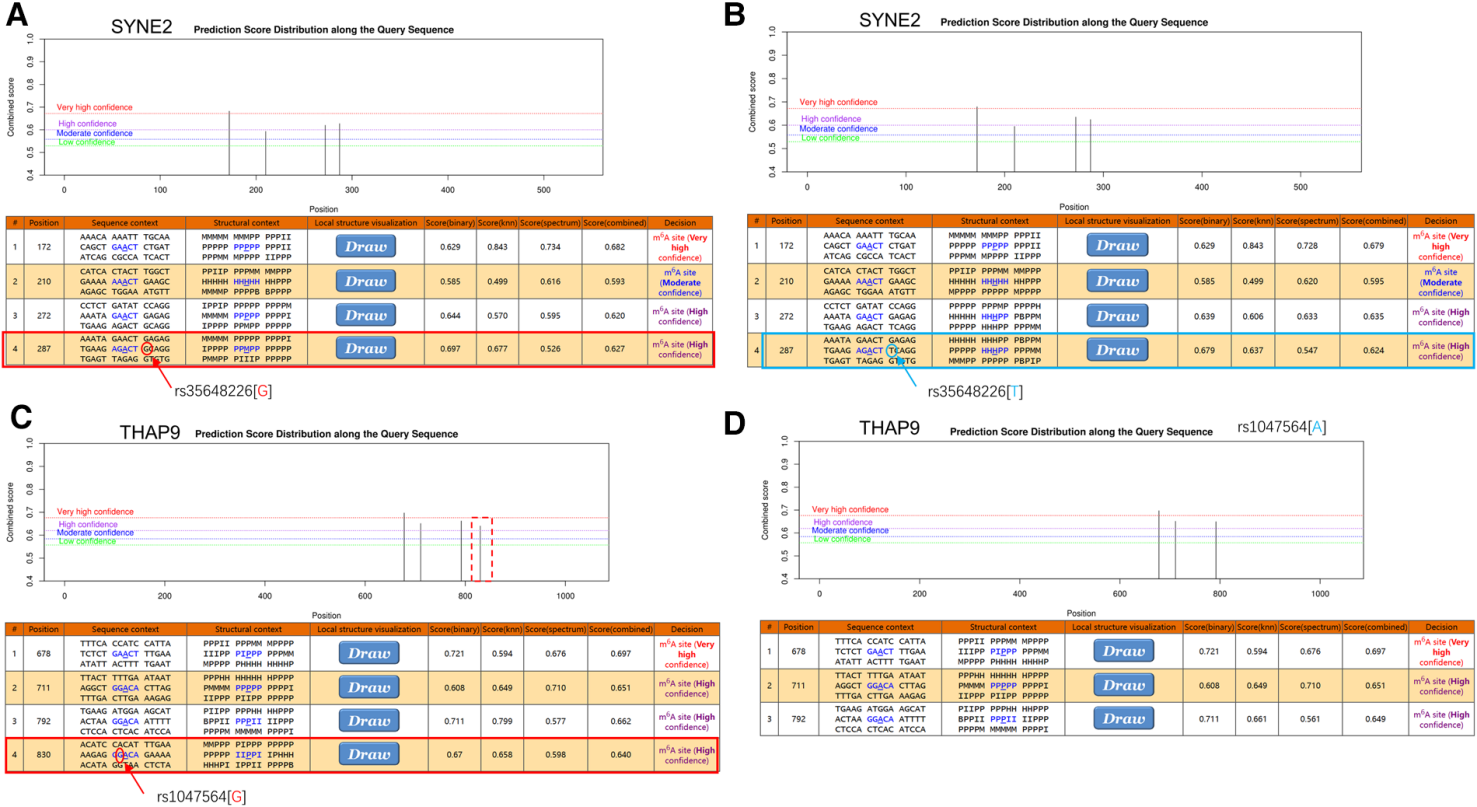
The m6A modification was predicted by using the genomic sequence of *SYNE2* and *THAP9* transcripts on website (http://www.cuilab.cn/scramp). (**A,B**) There were highly convincible m6A modification predicted peaks near *SYNE2*(rs35648226) and the G > T change was predicted to reduce the m6A level of *SYNE2*. (**C,D**) The red dashed box indicates the disappearance of the m6A modified peak near rs1047564 after inputting the altered sequence. The tracks sequentially show base position, FANTOM CAT, UCSC gene, transcription levels assayed by RNA-Seq on nine cell lines from ENCODE, DNase I hypersensitivity clusters, conservation and common SNPs.

**Figure 6:**
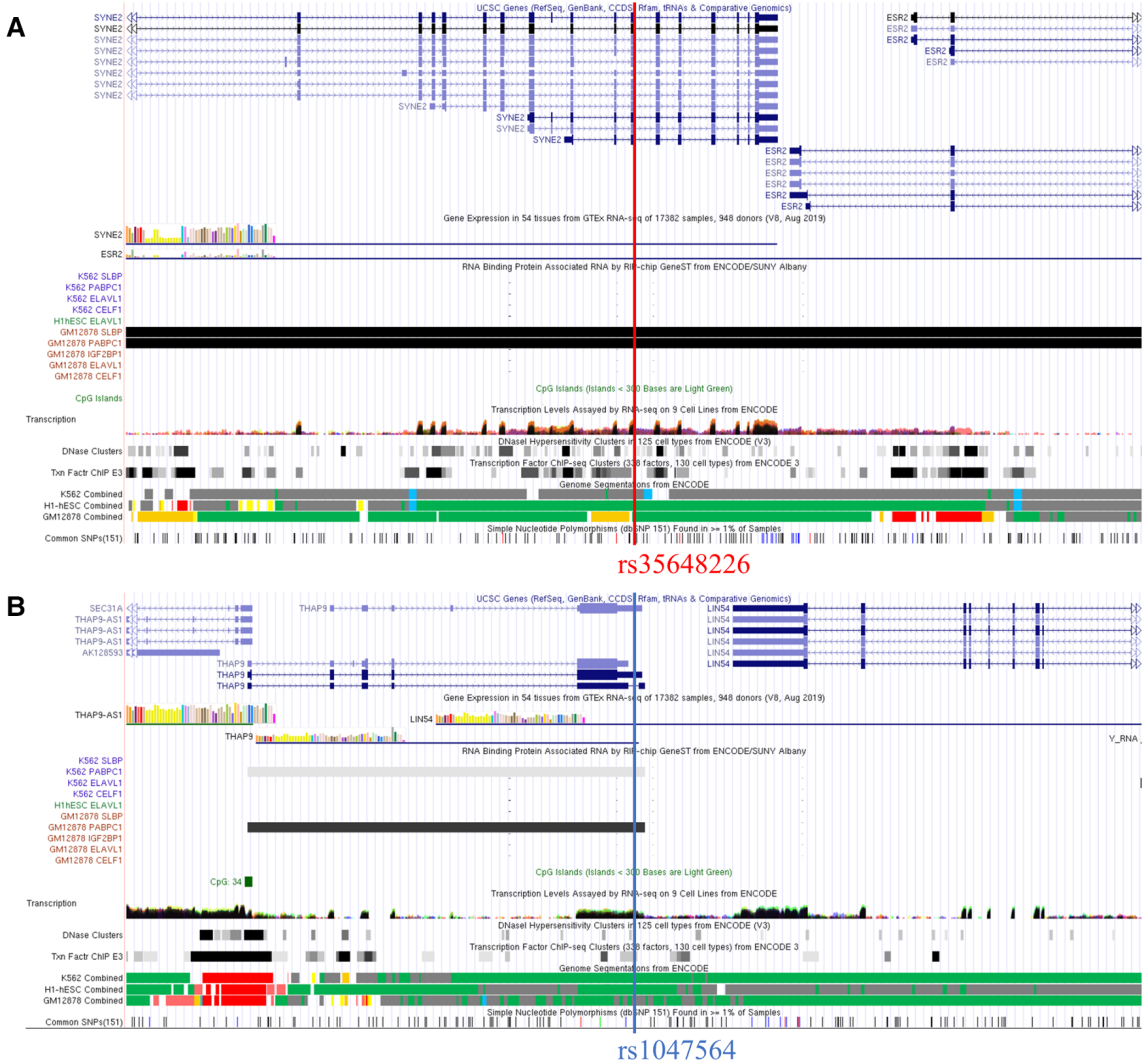
Integrative analysis of the potential function of rs35648226 and rs1047564 by querying UCSC. (**A**) The regional association map of the rs35648226 locus. The SNP rs35648226 was located in the *SYNE2* protein-coding region. RIP-chip GeneST from ENCODE/SUNY Albany data suggested that SLBP and PABPC1 might have a potential interaction with rs35648226. (**B**) The regional association map of the rs1047564 locus, which suggested that *PABPC1* might have a potential interaction with rs1047564.

To investigate the possible m6A methylation sites of *SYNE2* (rs35648226), *USP36* (rs900349) and *THAP9* (rs1047564), their transcript sequences or genomic sequences were predicted at the SRAMP website. According to the analysis results, the rs35648226 and rs1047564 that near to high convincible m6A-modified predicted peaks might have an influence on m6A modification. The rs35648226 is located in the CDS of *SYNE2* and is a G > T change. It is located 3 bp from a predicted m6A site annotated by SRAMP databases (Figure 5A,B), and the G > T change was predicted to reduce the m6A level of *SYNE2*. The Figure 5C,D showed that this rs1047564 G > A polymorphism highly reduced the *THAP9* m6A level. Previous studies (24–26) have suggested that alterations in the m6A level of RNA can impact the binding of RNA-binding proteins (RBPs), thereby influencing the gene expression level. Given that the SNPs identified in our study were found to affect the m6A level, we sought to further investigate which RBPs may be binding in the region where the SNPs are located. We next query the UCSC browser to analyze the potential function of the 2 m6A-SNPs. As is shown in Figure 6A, RIP-chip GeneST from ENCODE/SUNY Albany data suggested that *SLBP* and *PABPC1* might have a potential interaction with rs35648226 (*SYNE2*). The SNP rs1047564, located on the 3′UTR of the *THAP9* on chromosome 4, may interact with *PABPC1* (Figure 6B). Our findings imply that these two SNPs may be related to AF susceptibility by lowering the degree of m6A modification that regulates gene expression. Besides, rs35648226 and rs1047564 may also have an impact on the local gene expression by binding with the RBPs *SLBP* and *PABPC1*.

## Discussion

Studies have confirmed that m6A modification are important in cardiovascular diseases including myocardial infraction (15), heart failure (19), myocardial hypertrophy (16). But it's unclear whether m6A modification plays a role in AF risk and development. Here, our results reveal that m6A SNPs are associated with AF: First, we identify 105 m6A-SNPs that are associated with AF by analyzing data from AF GWAS and m6AVar database. Moreover, 7 m6A-SNPs show eQTL signals in atrial appendage. Besides, the results of our analysis reveal there are 3 m6A-SNPs corresponding to 3 genes are differently expressed in AF population. Further, 2 SNPs may influence AF risk by affecting m6A modification. These findings suggested that m6A-SNPs could be functional variants for AF.

As the most common post-transcription modification in eukaryotes, m6A modification can affect multiple physiological process and disease progression. m6A modification mediates cellular functions by regulating the mRNA metabolism process including nuclear export, stability, splicing, localization, and translation (27, 28). The m6A SNPs are usually adjacent to the methylation site. Therefore, the cellular functions involved in m6A modification will be influenced when m6A modification is altered by genetic variants (25). In addition, part of the m6A-SNPs are variants in UTR regions, which will affect transcriptional regulators and RNA binding protein. Besides, if the m6A-SNP is a missense variant, the amino acid composition as well as the secondary structure of the encoded protein would be changed. So, the m6A-SNPs have various functions which are worth being paid attention to. Thus, it's useful for exploring mechanism as well as the therapy strategy of AF to identify the m6A-SNPs associated with AF. Here, in our study, we find more than 100 m6A-SNPs are correlated with AF and our results suggest m6A-SNPs have vital roles in AF.

In the present study, we identified 105 m6A-SNPs associated with AF by analyzing the intersection of AF GWAS and m6Avar database, among which 7 showed eQTL signals in atrial appendage. Among these SNPs showing eQTL signals, rs2815301, rs1007654, rs900349 and rs28369993 were located in intron region. rs35648226 was located in CDS region. rs1047564 was located in 3′UTR region. rs1226590 was located in 5′UTR. We further prove the corresponding genes of the 3 m6A-SNPs (rs35648226, rs900349 as well as rs1047564) were differentially expressed by comparing the mRNA expression levels between AF patients and heathy controls, which suggests m6A-SNPs may be related to AF by influencing local gene expression. Moreover, *SYNE2* (rs35648226) and *THAP9* (rs1047564) were predicted to affect m6A modification of AF. When m6A modification is changed by the genetic variants, the biological process involved will be influenced as well. For example, if the variant is located in UTR, it will influence the transcriptional regulatory factors and RNA binding proteins. If the m6A-SNP is a missense mutation, not only will the m6A modification be affected, but the amino acid and secondary structure of the associated protein will be changed. Even if the variant is a synonymous one, it will make a difference in the RNA abundance transferred, which has an impact on the translation process (29). So, m6A-SNPs may affect the biological process by multiple ways. In our study, the AF-related m6A-SNP rs1047564 located in 3′UTR of the *THAP9* on chromosome 4 was predicted to influence the m6A modification and protein binding, thus correlated with AF, which was not reported. rs35648226 located in CDS region of *SYNE2* gene was also predicted to influence the m6A modification and protein binding*.* rs35648226 might have a potential interaction with *SLBP* and *PABPC1*, while rs1047564 might have a potential interaction with *PABPC1*, which suggest both of them are functional variants potentially by binding RBPs, which regulate the mRNA processing and metabolism. *THAP9* was concerned in cancer (30). *SYNE2* has previously reported to be related to AF (31, 32) and cardiac conduction (33). SNP rs1152591 located in an intron of *SYNE2* was clearly associated with AF. Here, we report the m6A-SNPs located in different genes associated with AF, which is of great importance in explaining the role of m6A modification in AF.

Furthermore, GO enrichment analysis found the most significant GO processes included multicellular organismal process (GO:0032501), cellular process (GO:0009987), metabolic process (GO:0008152), regulation of biological process (GO:0050789). The mechanism of AF is complicated and unclear. The current consensus is that electrical remodeling, inflammation, fibrosis as well as genetics are the major substrate for AF (34, 35). In our analysis, these m6A-SNPs are involved in biological regulation and signaling, which may regulate the electrical remodeling, inflammation, fibrosis (36).

In summary, we are the first to explore the relationship between m6A-SNPs and AF. In this study, we reported over 100 m6A-SNPs associated with AF which may contribute to AF risk and development and proved the potential function. Though we found strong evidence to support the connection between m6A-SNPs and AF, further studies especially molecular experiments on animal models and cell lines are needed to elucidate the underlying mechanisms and functions of these m6A-SNPs. However, this study still has some limitations, such as the lack of large sample support of patients with atrial fibrillation and experimental data. Therefore, the potential marker genes and SNPs identified in this study need to be further verified to provide solid evidence for clinical diagnosis and prevention.

## Data Availability

The datasets presented in this study can be found in online repositories. The names of the repository/repositories and accession number(s) can be found in the article.
